# What is New on Thyroid Cancer Biomarkers

**DOI:** 10.4137/bmi.s669

**Published:** 2008-04-29

**Authors:** Rosaria M. Ruggeri, Alfredo Campennì, Sergio Baldari, Francesco Trimarchi, Maria Trovato

**Affiliations:** 1 Sezione di Endocrinologia, Dipartimento Clinico-Sperimentale di Medicina e Farmacologia; 2 U.O. di Medicina Nucleare, Dipartimento di Scienze Radiologiche; 3 Dipartimento di Patologia Umana, University of Messina, Policlinico Universitario, “G. Martino”, Messina, Italy

**Keywords:** thyroid cancer, biomarkers, molecular signals, FNAC, tumorigenesis pathways

## Abstract

Thyroid cancer harbours in about 5% of thyroid nodules. The majority of them are well-differentiated cancers originating from the follicular epithelium, and are subdivided into papillary and follicular carcinomas. Undifferentiated carcinomas and medullary thyroid carcinomas arising from C cells are less common.

Although most thyroid nodules are benign, distinguishing thyroid cancer from benign lesions is crucial for an appropriate treatment and follow-up. The fine needle aspiration cytology (FNAC) allows the diagnosis of nature of thyroid nodules in the majority of cases. However, FNAC has some limitations, particularly in the presence of follicular lesions which can appear dubious in rare instances even at histology.

In an effort to improve diagnostic accuracy and offer new prognostic criteria, several immunohistochemical and molecular markers have been proposed. However, most of them have to be validated on large series before being used in routine practice.

## Introduction

### Thyroid tumours: a worldwide health problem

The expression “thyroid tumour” identifies a large variety of thyroid lesions which include well-differentiated and benign neoplasm or goitre nodules as well as either differentiated cancers of follicular origin or poorly differentiated and de-differentiated cancers, along with medullary and anaplastic carcinomas, all arising as a thyroid nodule (Sakamoto et al. 1983; Rosai et al. 1992; Baloch and LiVolsi, 2003).

Thyroid carcinomas account for about 90% of malignancies of the entire endocrine system and for 1% of human malignancies (Landis et al. 1998; Wong and Wheeler, 2000; Jemal et al. 2007). Annual incidence rate throughout the world, widely ranges from 0.5 to 13.6 cases per 100,000 inhabitant per year (Arnbjörnsson et al. 1986; Kolonel et al. 1990; Akslen et al. 1993; Colonna et al. 2002; Hernandez, 2003; Gomez Segovia et al. 2004).

Epidemiological reports of thyroid cancer are in striking contrast with statistical data on the epidemiology of cancerous diseases. In fact, data from the US through the Surveillance, Epidemiology, and End Results (SEER) program, and the European data from the GLOBOCAN 2002 project of the International Agency for Research on Cancer, indicate a progressive stabilization of the incidence and a decline of the mortality rates for most cancers from the year 2000 (Parkin et al. 1999; Greenlee et al. 2000; Hayat et al. 2007; Jemal et al. 2007). On the contrary, in worldwide population the thyroid cancer incidence has increased (Parkin et al. 2002; Hodgson et al. 2004; Davies and Welch, 2006; Albores-Saavedra et al. 2007; Jemal et al. 2007) and some studies have also reported increased mortality either in Europe or in USA (Howe et al. 2001; Parkin et al. 2002). However, the prognosis for thyroid cancer is good, so it accounts for rather few deaths (35,000 or 0.5% of all cancer deaths) with a 5 years survival rate of more than 90% (Mazzaferri and Kloos, 2001). Apart from patient age and histological type, prognosis is strongly determined by the stage of tumour at diagnosis and initial treatment. Therefore, a correct and early diagnosis is crucial for the outcome of the disease. In an effort to improve diagnostic and prognostic accuracy, specific and reproducible biomarkers of thyroid malignancy are necessary.

Aim of this review is an overview of well-established tests with a special emphasis for the effective role pertaining to new potential thyroid biomarkers in relying on the last knowledge of the molecular genetic events promoting thyroid tumorigenesis.

## Diagnosis of Thyroid Tumour and Identification of Malignancy

The identification of malignancy in a thyroid nodule is based on different clinical, biochemical, imaging and pathological tools.

Thyroid tumours arise almost invariably as a nodule, but the largest majority (about 95%) of thyroid nodules are benign (Utiger, 2005). Malignant thyroid nodules include papillary and follicular carcinomas, both originating from follicular epithelium, and a small number of undifferentiated cancer. Medullary thyroid carcinomas account for less than 5% of thyroid nodules and exhibit a specific circulating biomarker, i.e. calcitonin (CT), the levels of which clearly indicate its biological nature.

### Biological malignancy of thyroid tumours arising from follicular cells

The majority of follicular tumours (more than 90%) are well-differentiated, usually retain in varying degrees biological properties of normal follicular cells (i.e. iodine uptake; thyroglobulin synthesis), and are mainly divided into papillary (PTC) and follicular (FTC) carcinomas on the basis of the histological features (Rosai et al. 1992; Schlumberger, 2007).

PTC is the most common type of thyroid cancer (about 85%), with the highest rate of incidence in women (F: M ratio 4–8:1) and its incidence has increased in the last 20 years in different parts of the world (Haselkorn et al. 2000; Hodgson et al. 2004; Lang et al. 2007). The major risk factor for the development of PTC is a history of radiation exposure, especially during childhood, as proved by the consequences of the Chernobyl accident (Ron et al. 1995; Cardis et al. 2005; Schlumberger et al. 1999; Williams, 2000; Tronko et al. 2006). Several further risk factors including family history of thyroid cancer or benign thyroid disease, familial adenomatous polyposis of the colon and reproductive life in females may play a role (Navarro Silvera et al. 2005; Schlumberger, 2007). The possible influence of nutritional factors, including iodine nutrition and the exposition to chemical and physical contaminants of volcanic origin are presently under investigation in Sicily. (Frasca et al. 2008). PTC may arise as a single thyroid nodule; however, in at least 20% of cases, the evidence of a single nodule of PTC corresponds to a multifocal spread by a microscopic examination (Sherman, 2003). The biological malignancy of PTC is associated with a specific follicular cell-type, showing typical nuclear features, such as nuclear grooves and “ground glass” appearances (Rosai et al. 1992; Hermanek et al. 2002). PTC variants are classified on the basis of architectural and cytoplasmic features which may be associated with biological malignancy (Rosai et al. 1992; Hermanek et al. 2002; Trovato, 2007, p 191–202). Specifically, PTC variants include the classic, follicular, oncocytic, tall cells and diffuse sclerosing forms. Finally, PTC may be classified as microcarcinomas when measuring 10 mm or less in diameter. The outcome of PTC is variable depending mainly from patient’s age, size of tumour, pathological classification and presence of distant metastases (Mazzaferri, 2000; Pellegriti et al. 2004; Frasca et al. 2008). Lymph-node involvement is frequent and early in PTC, with specific localization to lymph nodes next to the trachea or windpipe, called the para-tracheal lymph nodes (Hermanek et al. 2002). Vascular invasion is rare, and distant metastases are observed in less than 10% of cases (LiVolsi, 1990; Grebe and Hay, 1997; Al-Brahim and Asa, 2006). Generally, the prognosis of PTC is excellent.

FTC is less frequent than PTC (5% to 15% of thyroid malignancies), and occurs mostly in women with a F:M ratio 2:1(Schlumberger, 2007). FTC usually presents as a solitary nodule irregularly bordered with invasive aspects in adjacent thyroid tissue. Its biological malignancy is linked to the pattern of tumour growth as well as to the morphology of tumour cells, and is based on the demonstration of vascular and capsular invasion. Through the cellular growth, the FTC architectural structure varies from well-formed follicles to a solid growth pattern, and different patterns may coexist. Considering the cellular morphology, FTC includes a variant with dark nucleus and eosinophilic cytoplasm and a less frequent variant with dark nucleus and oncocytic cytoplasm which has a higher grade of malignancy (Trovato et al. 2004). Through the degree of the invasion, the FTC is distinguished in minimally and widely invasive types, with a more favourable prognosis for the minimally invasive form. Apart from the histological type, the behaviour of FTC depends on patient’s age, size of tumour, extra-thyroidal extension and distant metastases (Hermanek et al. 2002; Schlumberger, 2007). FTC shows a major degree of aggressiveness with respect to PTC. In fact, FTC has a greater rate of recurrence and is more frequently associated with extra-glandular extension and distant metastases, preferring to spread through the blood stream (Rosai et al. 1992; Li Volsi, 1990; Grebe and Hay, 1997). FTC outcome is generally less favourable than PTC prognosis.

The undifferentiated carcinomas neither exhibit follicular or papillary patterns, nor retain the normal follicular functions, and are referred to as anaplastic carcinomas (ATC) (Grebe and Hay, 1997). ATC represents 1% of thyroid cancers and is one of the most aggressive human malignancies with a near 100% mortality. Characteristically, ATC grows and metastasizes quickly and does not respond to radioiodine therapy. Although histological features distinguish the ATC in two variants (spindle cells and giant cells), the biological malignancy is similar for both variants and the prognosis is bad because of the rapid aggressive course of the disease (Rosai et al. 1992; Pellegriti et al. 2002).

### Biological malignancy of thyroid tumours arising from parafollicular C-cells

Medullary thyroid carcinomas (MTC) derive from the parafollicular C cells of the thyroid that produce CT. It is less common (5% of thyroid cancers) than epitheliomas, and its management differs from that of follicular cell-type-specific cancers in many aspects (Wells and Franz, 2000). MTC may occur either in sporadic (75%) or familial form (25%) (LiVolsi, 1990). Commonly, the sporadic MTC type appears as a solitary nodule located in the middle third of the thyroid lobe, where C-cell concentration is intense. Instead, the heritable MTC type is multifocal, bilateral and associated to C cells hyperplasia. MTC exhibit a specific circulating biomarker, i.e. CT, produced by the tumour cells. In fact, basal CT levels are elevated virtually in all patients with the disease, and the highest values are observed in patients with the greatest tumour mass. The routine measurement of serum CT has been proposed in the diagnostic work-up of all patients with thyroid nodules in order to allow the pre-operative diagnosis of MTC even in a sub-clinical phase (Pacini et al. 1994; Vierhapper et al. 1997; Elisei et al. 2004). Clinical trials have demonstrated that MTC diagnosed through CT screening had a significant lower stage at diagnosis and a better prognosis after a 10-year period of follow-up. The measurement of plasma CT levels, both basal and after a provocative test (i.e. pentagastrine test), allows the identification of MTC at a pre-clinic stage and in the family members of patients with inherited MTC. Furthermore, RET germinal mutations are employed to identify inherited MTC, both isolated and occurring in MEN (Elisei et al. 2007).

The aggressiveness of MTC depends on the proliferation and growth rate of the tumor. Although there are several histological variants of MTC, most of them have no importance in determining the biological aggressiveness of MTC.

MTC metastasizes to regional lymph nodes, but may invade blood vessels and distant metastases are present at diagnosis in up to 20% of cases (Rosai et al. 1992; Wells and Franz, 2000). Clinical prognostic factors in MTC include age at diagnosis, male gender, tumour size and initial extend of the disease (Pellegriti et al. 2003).

## Diagnostic Approaches to Thyroid Cancer: Which Biomarkers May be Useful to Recognize the Malignancy

Diagnostic markers are constantly investigated to overcome the difficulties to distinguish benign from malignant thyroid lesions.

In an effort to improve diagnostic accuracy, many immunohistochemical and molecular biomarkers have been proposed, but the clinical implications have been demonstrated only for some of them. Beyond their diagnostic value, these biomarkers should be able to offer prognostic criteria, and may also play a role in detecting persistent or recurrent disease as well as in choosing the therapeutic strategies. Particularly, they may improve the sensitivity and accuracy of FNAC, and so may contribute to reduce the frequency of surgical procedures by identifying those patients with benign lesions who do not require surgical excision.

Based on the methodology applied to recognize the diagnostic traits, it is possible to distinguish cytological and histological thyroid biomarkers, such as immunohistochemical and molecular biomarkers.

### Cytological evaluation

Fine needle aspiration cytology (FNAC), routinely performed in the clinical practice, allows the diagnosis of the nature of thyroid nodules with an accuracy of over 90% (Hamburger, 1994; Gharib, 1996; Gharib and Papini, 2007).

**Figure 1:**
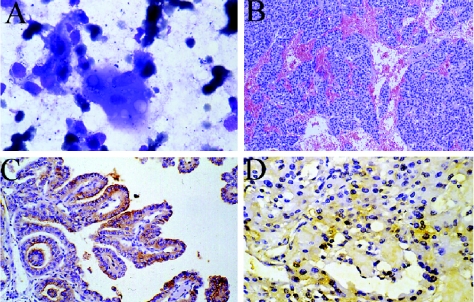
**A.** Cytological smears of papillary thyroid carcinoma. MGG stain, obj ×40. **B.** Histological features of follicular thyroid carcinoma. E-E stain, obj ×10. **C.** c-met immunoreaction in papillary thyroid carcinoma, obj ×20. **D.** MDR2 expression in medullary thyroid carcinoma, obj ×20.

In fact, FNAC is the most effective test adopted for an accurate diagnosis of PTC, ATC and MTC nodules in the pre-surgical stage. The PTC identification by FNAC is assessed primarily on the basis of nuclear changes (Baloch and LiVolsi, 2005; Das et al. 2004; Das, 2005; Trovato, 2007, p 191–202). PTC nuclei are large, elongated, overlapping and crowding with irregular outlines, intranuclear and cytoplasmic pseudo-inclusions, ground-glass appearance, multiple small nucleoli and deep grooves (Fig. 1A). Instead, the observation of spindle or giant cells in cytological smears of FNAC allows to identify ATC, while the detection of monomorphic, large polygonal cells related to the diffused neuroendocrine cell system leads to a diagnosis of MTC (Trovato, 2007, p 191–202).

However, FNAC has some limitations (Hamburger, 1994; Gharib, 1996; Gharib and Papini, 2007). FNAC is less useful to diagnose FTC, because the major criteria for differential diagnosis are the capsular and vascular invasion which cannot be seen on cytological smears. Therefore, the FTC diagnosis is assessed only after surgery through the histological evaluation (Baloch et al. 2002; Louvel and Vielh, 1999; Deveci et al. 2006; Haugen et al. 2002). When a follicular or Hürthle cell proliferation is displayed, cytology cannot differentiate the carcinoma from its benign counterpart, and the histological verification of the lesion is indicated. Further, FNAC interpretation is difficult when papillary or medullary cancer, that usually are easy to diagnose for an expert cytologist, do not exhibit the typical features but the cellular pattern is atypical; in such cases the lesion may be misinterpreted.

Obviously, FNAC has a diagnostic value when the cellularity is adequate. Inadequate FNAC (15%–20%, in different series) should be repeated, and surgical treatment may be recommended.

#### Histological examination

Histological evaluation improves the sensitivity and accuracy of FNA overcoming the difficulties to distinguish benign from malignant thyroid lesions.

Specially, histological examination is required to achieve the diagnosis of FTC, which is composed by follicular cells with dark nuclei and cytoplasms being eosinophilic in most of FTC and oncocytic in the Hürthle cells variant. In fact, in both cases the diagnosis of malignancy is based on the demonstration of vascular and capsular invasions, which are absent in follicular and oncocytic adenomas (Louvel and Vielh, 1999; Haugen et al. 2002) (Fig. 1B). Furthermore, histological examination allows to distinguish the minimally invasive (encapsulated) and widely invasive cancer types thought the pattern of growth of cancerous follicular cells.

However, the diagnosis of FTC may be difficult even at the final histological examination, when the capsular and vascular invasion is minimal.

### Immunohistochemical biomarkers

The need to add immunohistochemical biomarkers to the cytological and histological investigations above illustrated derives from the difficulties of a differential diagnosis in borderline cases (e.g. carcinomas with atypical cellular pattern, follicular or oncocytic lesions, such as follicular adenomas and carcinomas; Hürthle cells adenomas and carcinomas; follicular variant of PTC). Immunohistochemistry is an important technique able to recognize specific proteins on cytological or histological specimens. All thyroid diagnostic immunomarkers share the ability to be expressed when the carcinoma appears. However, the expression of the currently known biomarkers is strongly variable in sensitivity and specificity and then, to date, none of them have been routinely adopted in cytological and/or histological diagnostic procedures.

Mainly, the immunohistochemical biomarkers are employed to reach an accurate diagnosis of PTC and MTC, whereas there are no valued biomarkers to facilitate the diagnosis of FTC.

Immunohistochemical investigation of PTC had identified several biomarkers, showing varying degrees of sensitivity and specificity. The biomarkers that up to date appear to yield the higher sensitivity and specificity include Hector Battifora mesothelial cell 1 (HBME-1), high molecular weight cytokeratin 19 (CK19), galectin-3, c-met.

HBME-1 is a monoclonal antibody generated against the microvillous surface of mesothelial cells of mesothelioma. It has been reported to be reactive in most cases of papillary thyroid carcinoma, and absent in benign thyroid lesions (Sack et al. 1997; de Matos et al. 2005). So, HBME-1 is quite specific marker for PTC. However, this biomarker shows a poor sensitivity, because PTC lesions with oncocytic cells are uncreative (Sack et al. 1997), and a negative result for HBME-1 does not preclude the diagnosis of carcinoma.

CK19 is a high-molecular-weight cytokeratin showing a strong sensitivity but poor specificity for PTC. In fact, the immunoexpression of CK19 is assessed to support the PTC diagnosis in borderline lesions because of its widespread reactivity (Ramphael et al. 1995; Rorive et al. 2002), but positive immunoreactivity for CK19 of varying degrees has been observed in normal thyroid tissue around PTC and in benign thyroid lesions, thus limiting the usefulness of this marker (Cheung et al. 2001).

Galectin-3, a member of the beta-galactoside binding family of lectins, is strongly expressed in PTC, mostly in the classic variant. Immunoreactivity for galectin-3 was also reported in a number of FTC, and it has been considered of some value in differentiating between benign and malignant follicular lesions (Gasbarri et al. 1999; Saggiorato et al. 2004; Nucera et al. 2005). Therefore, immunocytochemistry with anti-Galectin-3 antibodies has been proposed as an auxiliary procedure to conventional cytology in the diagnostic work-up of thyroid nodules (Bartolazzi et al. 2001; Saggiorato et al. 2004; Collet et al. 2005). However, the specificity of Galectin-3 is poor because of its reactivity in benign goitre and thyroditis lesions (Orlandi et al. 1998; Mehrotra et al. 2004). In addition, other studies have found that galectin-3 is not a sensitive indicator of thyroid cancer because of the possibility of false-negative results in specific cases, such as Hürthle cell proliferation or minimally invasive follicular carcinomas (Oestreicher-Kedem et al. 2004; Mehrotra et al. 2004).

Based on the literature, none of the above discussed biomarkers appears to be reliable in identifying all malignant thyroid lesions in highly specific and sensitive manner. As compared with the use of single biomarker, the combination of two or three markers may represents a more accurate immunohistochemical approach in the differentiation of malignant tumors from their benign counterparts, especially in controversial categories (Asa, 2005; de Matos et al. 2005; Prasad et al. 2005; Saggiorato et al. 2005; Nasr et al. 2006).

The c-Met proto-oncogene, localized on the long arm of chromosome 7, encodes for a tyrosine-kinase receptor, namely, c-met. Its high affinity ligand is a pleiotropic mesenchyme-derived cytokine, the hepatocyte growth factor (HGF). HGF/c-met interaction can promote different responses (i.e. scattering, growth and morphogenesis) in epithelial cells through activation of several pathways. Among thyroid cancers, PTC is associated with marked over-expression (up to 100 folds) of the c-met proto-oncogene, which, instead, is rarely expressed in other thyroid tumors, including FTC, ATC and MTC (Di Renzo et al. 1995; Oyama et al. 1998; Trovato et al. 1998). Over-expression of c-met has been found in 75%–100% of PTC, regardless of the histological variants; it was not associated with gene amplification or rearrangements, and the protein produced by the tumor cells did not show structural alterations. Our group have demonstrated for the first time the immunohistochemical co-expression of HGF and c-met in PTC cells (up to 80% of PTC studied), raising the possibility of met activation through an autocrine loop (Trovato et al. 1998). Moreover, we have correlated the expression of HGF/c-met with that of STAT3, one member of the Signal Transducers and Activators of Transcription, which is known to mediate the morphogenetic effects of HGF/c-met (Boccaccio et al. 1998). We have demonstrated that the whole morphogenetic pathway HGF/c-met/STAT3 is over-expressed in PTC (Fig. 1C), and is highly specific for this type of thyroid malignancy (100% of PTC tested; none of the FTC or ATC), suggesting that such autocrine signalling pathway may be relevant for the establishment of the papillary phenotype (Trovato et al. 2003). Met and/or HGF and/or STAT3 are not expressed in normal thyroid tissue, but in our hands, a number of totally benign lesions were HGF^+^/c-met^+^ without being STAT3^+^ (Trovato et al. 1998; Trovato et al. 2003). Only a subset of follicular adenomas expressed the whole c-met/HGF/STAT3 pathway, and we have hypothesized that these adenomas may progress to PTC (Trovato et al. 2003). Since the expression of HGF, c-met and STAT3 appears to be a typical trait of almost every case of PTC, including all the histological variants, these molecules may be included among the best candidates to the role of PTC markers. Clinical trials on larger series are required to verify their usefulness either in the diagnosis of PTC or in the recognition of PTC precursor lesions (Trovato et al. 1998; Trovato et al. 2003).

CT is produced from C cells and therefore positive immunoreaction of malignant cells with anti-CT antibodies allows an unequivocal diagnosis of MTC. Despite the CT expression being highly specific, its immunoreaction is not constantly detected in MTC. Chromogranin is a more sensitive immunomarker with respect to CT and its use is foreseen in each diagnostic algorithm for MTC (Rosai et al. 1992; Wells and Franz, 2000).

### Molecular biomarkers

The thyroid molecular biomarkers correspond to genetic mutations arising in malignant thyroid cells and recognizable by the molecular biology techniques. Several molecular alterations (mutations and/or gene rearrangements) have been described in thyroid malignancies, and it has been demonstrated that different genes and signalling pathways are involved in the development of PTC and FTC as well as MTC. The expression of each molecular marker may be studied on frozen specimens of the neoplastic tissue by using PCR techniques.

The more common genetic abnormalities found in PTC are the radio-induced RET/PTC rearrangements and the mutations of BRAF and RAS genes, while PAX8/PPARγ fusion gene and the loss of heterozigosity (LOH) on 3p and 7q loci as well as RAS mutations are frequently encountered in FTC.

BRAF is a serine—threonine kinase involved in the mitogen-activated protein kinase (MAPK) pathway. Mutations of the BRAF gene, located on the long arm of chromosome 7, represent the most common genetic alteration in PTC and seem very close to this type of cancer, because they are not found in other histotypes (Nikiforova et al. 2003; Trovisco et al. 2005). BRAF point mutations at 600 (BRAF^V600E^) and—less frequently—at 599 and 601 locations, resulting in constitutive kinase activation, have been detected in about 26%–69% of sporadic PTCs of adults (Soares et al. 2003; Trovisco et al. 2004; Cohen et al. 2003; Trovisco et al. 2005; Moretti et al. 2006). Recently, a BRAF rearrangement by paracentric inversion of chromosome 7q followed by the fusion between AKAP9 and BRAF genes has been recognised in a subset of radiation-induced PTC (Ciampi et al. 2005). BRAF mutations are strongly associated with the classic variant of PTC, displaying the typical nuclear features and the papillary architecture, although they have been also reported in tall cell or columnar cell variants (Nikiforova et al. 2003; Begum et al. 2004; Trovisco et al. 2004). In several studies, the presence of BRAF mutations has been associated with older age of patients, more advanced stage of the disease at presentation and higher frequency of recurrence and/or metastases (Namba et al. 2003; Nikiforova et al. 2003; Vasko et al. 2005; Riesco-Eizaguirre et al. 2006). Moreover, a significant incidence of BRAF^V600E^ mutation has been found in undifferentiated thyroid cancers, suggesting that ATC may arise from more typical forms of PTC and that BRAF signalling may be functionally relevant in tumor progression (Nikiforova et al. 2003; Begum et al. 2004; Soares et al. 2004; Trovisco et al. 2004; Santarpia et al. 2008). However, the relationship between BRAF mutations and more aggressive tumour behaviour have not been confirmed in a number of other studies (Trovisco et al. 2005; Fugazzola et al. 2006), and a high frequency of BRAF mutations has been reported in microcarcinomas with an excellent prognosis (Kim et al. 2005; Fugazzola et al. 2006; Rodolico et al. 2007). In a very recent study in Sicily (Frasca et al. 2008) BRAF^V600E^ mutation was found in 52% classical PTC and in 26% in the tall cell variant. The mutation was found in macro-carcinomas more frequently than in microcarcinomas, is associated with extra-thyroid invasion and is positively correlated with Matrix Metalloproteinases 9 expression. BRAF mutation was found more prevalent in the Eastern part of the Island thus suggesting a possible environmental influence correlated to the volcanic nature of the large area surrounding Mount Etna. These data, taken together, underline how the BRAF mutations accompany the development of PTC, showing its relevant significance in the PTC tumorigenesis. Thus, search for BRAF mutations might be used in clinical practice to reach the cytological diagnosis of PTC and to select patients for a more aggressive initial treatment (Xing et al. 2004; Domingues et al. 2005; Pizzolanti et al. 2007; Sapio et al. 2007). Methods for rapid detection of these mutations on FNAC samples have been already developed (Xing et al. 2004).

The second most common genetic alteration described in PTC is the RET/PTC rearrangement. RET is a proto-oncogene, located on chromosome 10q11.2, encoding for a transmembrane tyrosine-kinase receptor (Santoro et al. 2004). The rearrangements known as RET/PTC lead to the constitutive activation of the tyrosine kinase receptor RET, that activate the RAS–RAF–MEK and other signalling cascades, thus promoting cell growth and transformation (Grieco et al. 1990). The most common RET/PTC rearrangements, namely RET/PTC1 and RET/PTC3, result in an intrachromosomal inversion of the long arm of chromosome 10 leading to the fusion of RET with the H4/D10S170 or RFG/ELE1 genes, respectively (Grieco et al. 1990; Santoro et al. 1994). Such gene rearrangements are more prevalent in pediatric patients and in PTCs developed following radiation exposure (Nikiforov et al. 1997; Nikiforov et al. 1999). In fact, in the adult population, the incidence rate of RET rearrangements is about 15%–20% of PTC, while in post-Chernobyl PTCs they have been found in up to 87% of cases. RET/PTC rearrangements are restricted to PTC, including both conventional PTC and oncocytic and diffuse sclerosing variants, thus representing a marker for this type of thyroid tumour. For this reason, RET/PTC detection in FNAC specimens have been proposed as a diagnostic adjunctive tool in the cytological evaluation of thyroid nodules (Salvatore et al. 2004; Domingues et al. 2005; Pizzolanti et al. 2007; Sapio et al. 2007). However, its specificity has been questioned because of the identification of RET/PTC in non-neoplastic follicular cells in Hashimoto’s thyroiditis (HT), oncocytic tumors, and other benign lesions, and this expression variability should be taken into account for the molecular diagnosis of thyroid lesions (Cinti et al. 2000; Elisei et al. 200; Nikiforova et al. 2002; Rhoden et al. 2004). Moreover, these rearrangements reveal the appearance of PTC cells in thyroid gland and seem deserved of prognostic significance. Despite this, the vast majority of the RET/PTC positive tumors are stage I at presentation. (Nikiforov et al. 1999; Viglietto et al. 1995). Finally, RET/PTC rearrangements are a common finding in papillary microcarcinomas, thus suggesting that they represent an early event in the tumorigenesis.

Another type of genetic alteration found in PTC is RAS point mutations involving specific regions (codons 12, 13 and 61) of the three RAS oncogenes namely, H-RAS, K-RAS, and N-RAS. RAS mutations are found not only in PTC, but also in FTC and ATC, and this genetic abnormality seems closer to the follicular cancers rather than to classic PTC. The incidence of RAS mutations is variable in these different histotypes, ranging from 0%–50% in PTC, 14%–62% in FTC, and 0%–60% in ATC. Among PTC, the follicular variant shows the highest prevalence of RAS mutations, as well as a lower prevalence of BRAF mutations and RET/PTC rearrangements, in respect to the conventional PTC and the other variants (De Micco, 2003; Zhu et al. 2003; Giordano et al. 2005). Moreover, RAS mutations are encountered even in benign follicular adenomas with a frequency ranging from 0%–85%. Nevertheless, a higher rate of RAS mutations in malignant rather than in benign thyroid tumours has been observed. These evidences suggest that RAS mutations represent the earliest events in cancer progression along the malignant pathway leading to FTC and ATC (Namba et al. 1990). It has been proposed that activating mutations in RAS oncogenes could be related to chromosomal and genomic instability, thus predisposing follicular cells to the accumulation of additional molecular abnormalities. However, the mutations of RAS have no diagnostic significance to distinguish the follicular adenoma from FTC (Vasko et al. 2003; Zhu et al. 2003).

PAX8/PPARγ fusion gene and the loss of heterozigosity (LOH) on 3p and 7q loci may represent potentially useful molecular biomarkers of FTC. The paired box 8 (PAX8) gene, located on 2q13 chromosome, and peroxisome proliferator-activated receptor γ (PPARγ) gene, located on 3p25 chromosome, encode thyroid-specific transcription factors. Pax-8 is a member of the Pax family of transcription factors, and is essential in the development of thyroid follicular cells and regulation of thyroid-specific gene expression. Mutations of PAX8 have been identified in cases of congenital hypothyroidism caused by thyroid dysgenesis (Macchia et al. 1998). PPARγ is a member of the nuclear hormone receptor superfamily that includes thyroid hormone, retinoic acid and androgen and estrogen receptors. The two genes are involved in a chromosomal traslocation leading to the fusion of the exons 7, 8 and 9 of PAX8 with exon 1 of PPARγ. The PAX8/PPARγ fusion protein includes the DNA binding domains of PAX8 and the PPARγ nuclear receptor domains. PAX8-PPAR-gamma disrupts normal transcriptional function of both transcriptional factors in thyroid follicular cells by inhibiting PPARγ trascriptional activation and dysregulating the PAX8 pathways (Kroll et al. 2000; Gregory Powell et a Gregory Powelll, 2004; Ay et al. 2006). This type of mutation is frequently observed in FTC and seems to be involved in FA to FTC progression. In fact, only 10% of FA expresses PAX8/PPARγ fusion gene such as the ATC expresses infrequently this rearrangement (Nikiforova et al. 2002; Cheung et al. 2003; Nikiforova et al. 2003; Lacroix et al. 2004). In line with these results, the expression of PAX8/PPARγ may be supposed as a marker of the well-differentiated FTC and its absence as a marker of tumour progress to rapidly fatal forms of thyroid carcinomas. Although the diagnosis of FTC is often problematic, the practical diagnostic use of this biomarker is limited because few clinical trials have been carried out to confirm the effectiveness of this biomarker (Puxeddu and Fagin, 2001; Nakabashi et al. 2004).

LOH on chromosomes 3p and 7q is frequently observed in the early steps of follicular tumoral transformation (Grebe et al. 1997; Ward et al. 1998; Zhang et al. 1998; Trovato et al. 1999; Trovato et al. 2004). The progression of the FA towards FTC is underlined through the appearance of both 3p and 7q LOH. LOH of chromosome 3 is more significantly found in FTC and follicular adenomas involving specific minimal common deleted regions corresponding to 3p25.3 and 3p21.2 loci, respectively (Trovato et al. 1999). The highest rate of LOH on chromosome 7 is located on 7q21.2 locus. Characteristically, LOH on 7q21.2 is specific for cell type because it involves only the benign and malignant thyroid lesions made up of dark nucleus and eosinophilic cytoplasm cells. Furthermore, to show specificity for the cellular phenotype, the 7q21.2 LOH increases along with neoplastic transformation reaching a 100% of expression in FTC correlating with thyroid gland volume and the presence of multiple lesions (Trovato et al. 2004). Thus, the 7q21.1 LOH has been proposed as a diagnostic key to assist pathologists in the task of distinguishing FTC from benign thyroid lesions. In fact, all suspected FTC lesions showing a prevalence of dark nucleus and eosinophilic cytoplasm cells may be included among benign lesions if they do not express LOH on 7q21.2 (Trovato et al. 1999; Trovato et al. 2004).

In MTC, the malignant transformation of C cells is characterized by the appearance of specific defects of the RET gene. RET germ-line mutations are inheritable occurring in hereditary MTC, whereas RET somatic mutations arise only in the context of the neoplastic C cells of the sporadic MTC. These differences have a practical application because the recognition of each RET germ-line mutation leads to an unequivocal diagnosis of hereditary MTC. Furthermore, RET germinal mutations are employed to identify inherited MTC, both isolated and occurring in MEN (Komminoth et al. 1995; Fink et al. 1996). Specifically, the germinal mutations involving the exons 10 and 11 are associated with MTC appearing in MEN 2A, while the germinal mutation of the exons 16 is linked to MTC arising in MEN 2B. To screen the isolated familial form of MTC, several germinal mutations located in exons 10,11,13,14 and 15, respectively, are individualized (Marsh et al. 1997; Chiefari et al. 1998; Kebebew et al. 2000; Iacobone et al. 2002).

## Prognostic Evaluation of Thyroid Cancer: What May Indicate Biomarkers

Different scoring systems, based on multiple regression analysis of combined predictive factors, have been proposed for stratifying patients with thyroid cancer into low and high risk prognostic groups. The most used scoring system for predicting survival is the TNM staging system in which T indicates the extension of primary tumour, N is for condition of regional lymph nodes, and M for presence of distant metastases. The most recent (2002) version of TNM has underlined the importance of the age of patients together with the histological type in the behaviour and prognosis of FTC and PTC (Greene et al. 2002; Hermanek and Sobin, 2002; Lang et al. 2007a; Lang et al. 2007b). Two important changes made in respect to the previous edition need to be addressed. First, all tumors ≤2.0 cm in size but limited to the thyroid gland are now classified as T1, while only tumors ≤1.0 cm were previously classified as T1. This might lead to under-treatment of some patients, exposing them to a higher risk of recurrence because of a less aggressive initial treatment (i.e. lobectomy, as performed in some centers for T1 tumors). Second, nodal involvement causes a shift towards a higher stage and less favourable prognosis also in tumors that, because of small size and patient age, usually have favourable outcome. Available evidence, however, indicates that lymphnode metastases have a limited prognostic impact on overall survival (Hughes et al. 1996).

Other scoring systems include AMES, AGES and MACIS, the Ohio State University Scoring System. The AMES system is based in the combination of Age, distant Metastases, Extend and Size of the primary tumor. On this basis, patients with PTC and FTC are subdivided in a low-risk group (age ≤40 ys men, 50 ys women; no distant metastases; older patients with intra-thyroid extension or minor capsular invasion and tumor size ≤5 cm) and a high-risk group (age ≥40 ys men, 50 ys women; distant mestastases; extra-capsular extension and size ≥5 cm of the primary tumor) (Cady, 1998). The AGES system was developed at the Mayo Clinic in 1987 and included the following variables: Age, Grade (according to the Broder’s classification), Extent (both local invasion and distant metastases) and Size of the primary tumor. In 1993, this scoring system was reviewed by the authors, excluding tumor grade and including distant Metatases, Age, Completeness of surgery, Invasion of the extra-thyroidal tissues and Size of the tumor (MACIS) (Hay et al. 1993). The Ohio State University Scoring System differ from the other systems because it not include age at diagnosis among variables. It distinguishes among patients with PTC and FTC, 4 categories according to tumor size, neck lymph-node metastases, multifocality, local invasion, and distant metastases (Mazzaferri and Kloos, 2001). The choice of the scoring system is mainly based on individual experiences. No significant difference was found in a comparative study of the TNM, AMES, AGES and MACIS systems. None of the many systems proposed has shown clear advantages in predicting thyroid cancer outcome (Brierley et al. 1997; Loh et al. 1997; Voutilainen et al. 2003; Lang et al. 2007a; Lang et al. 2007b). The TNM classification, however, remains the most widely used because it provides a shorthand method to describe the extent of the disease and also because it uses parameters commonly used by oncologists for other organ tumors.

Prediction of thyroid cancer outcome is commonly based on circulating thyrogobulin measurement in the complete absence of eutopic thyroid tissue. It is well established that thyroglobulin plays a reliable role in the monitoring of the well-differentiated carcinoma course, after total thyroidectomy. In fact, thyroglobulin assay allows detecting evidence of persistent or recurrent follicular thyroid cancers (Pacini et al. 2006; Castagna et al. 2008).

The other commonly used serum marker of neoplastic diseases is the CT that sets the clinical stage of MTC recurrence following the total thyroidectomy (Hermanek et al. 2002; Loh et al. 1997; Jukkola et al. 2004; Akslen and LiVolsi, 2000; Schlumberger, 2004; Whitley and Ain, 2004; Kebebew et al. 2000). Moreover, immunohistochemical evaluation of CT expression in tumor specimens has also a prognostic value, because MTC tumors with a negative or scarce immunostaining for CT appear to be more aggressive.

New prognostic biomarkers are currently searched and tested. However, up to date it is unclear if any of these biomarkers might be more accurate than the currently used clinical staging systems.

Met over-expression in PTC has been correlated with early stages of disease and a better outcome. In fact, cases of poorly-differentiated PTC showed decreased expression of met. In a study from Belfiore and co-workers (Belfiore et al. 1997) it has been reported that negative/low met expression was predictive of distant metastases, and its clinical use was proposed to identify high risk patients.

BRAF mutations in PTC seems to correlate with a poor outcome of the disease (Namba et al. 2003; Nikiforova et al. 2003; Oler et al. 2005; Vasko et al. 2005; Xing et al. 2005; Riesco-Eizaguirre et al. 2006; Giannini et al. 2007; Kebebew E et al. 2007; Lupi et al. 2007; Rodolico et al. 2007), and a recent study (Frasca et al. 2008) suggests higher aggressiveness even in the microPTC. However, as noted above, a clear relationship between BRAF mutations and aggressive tumour behaviour has not been demonstrated in all studies (Puxeddu et al. 2004; Kim et al. 2005; Trovisco et al. 2005; Fugazzola et al. 2006; Sapio et al. 2006; Abrosimov et al. 2007; Durante et al. 2007; Mitsiades et al. 2007), and the predictive value of BRAF mutations remains uncertain.

The plasminogen activator (PA) system, which consists of two serine proteases, the urokinase PA (uPA) and the tissue-type PA (tPA), their two serpin inhibitors (PAI-1 and PAI-2), and the glycolipid-anchored receptor for the uPA (uPAR), is involved in cancer progression, since it enhances both distant metastasis and direct invasion. In particular, the uPA system has been implicated in neo-vascularization and in remodelling of the extracellular matrix, enhancing both cell proliferation and migration and modulating cell adhesion. Consistent with their role in cancer progression, high levels of expression of uPA, uPAR and PAI-1 correlate with poor patient prognosis and outcome in several human cancers. For this reason, the uPA system components have been proposed as new prognostic markers for many cancer types, such as breast cancer (Dass et al. 2007; Duffy, 2004; Duffy and Duggan, 2004). In the late few years, data regarding the expression of the uPA system in thyroid cancer has yielded valuable insights. It has been demonstrated that both human thyroid carcinoma-derived cell lines and most thyroid carcinomas (PTC, FTC and ATC) overexpress uPA, uPAR and PAI-1 (Ito et al. 1996; Packman et al. 1996; Zanetti et al. 1998; Smith et al. 1999; Kim et al. 2002; Chu et al. 2004; Ulisse et al. 2006). Furthermore, the highest levels of expression were found in anaplastic carcinomas (Horvati Herceg et al. 2006), in well-differentiated carcinomas in which extra-thyroidal invasion or distant metastases had been present (Horvati Herceg et al. 2006; Ulisse et al. 2006) and in PTC whose size exceeded 1 cm in diameter (Horvati Herceg et al. 2006; Ulisse et al. 2006). Taken together, these data suggest that the PA system components have prognostic relevance in thyroid malignant tumors, as proven in other malignancies, and may represent candidate molecular biomarkers of this type of cancer.

In MTC, a relationship between somatic RET mutations and bad prognosis has been described. Recently, Elisei et al. demonstrated that the presence of a somatic RET mutation correlates with a worse outcome of MTC patients, not only for the highest probability to have persistence of the disease, but also for a lower survival rate in a long-term follow-up. More interestingly, the presence of a somatic RET mutation correlates with the presence of lymph node metastases at diagnosis, which is a known bad prognostic factor for the definitive cure of MTC patients (Elisei et al. 2007).

The poor outcome and prognosis of MTC is also related to the highly chemoresistance of this malignant neoplasia. Chemotherapy failure has been ascribed, at least in part, to the overexpression by MTC cells of the multidrug resistance proteins (MDRs), especially MDR1 and MDR2, transmembrane glycoproteins that antagonize intracellular accumulation of cytotoxic agents (Yang et al. 1991; Massart et al. 1995; Massart et al. 1996a; Massart et al. 1996b; Zatelli et al. 2005; Ruggeri et al. 2006). The expression and function of MDR1 and MDR2 have been demonstrated to depend on cyclooxygenase (COX)-2 levels, which are found elevated in many human cancers (Taketo, 1998a; Taketo, 1998b; Williams et al. 1999; Patel et al. 2002; Sorokin A, 2004). In a recent study we evaluated the immunoexpression of MDR2 and COX2 in MTC samples and correlated the expressions of these two proteins with pTNM and clinical stages. We demonstrated that MDR2 is constitutively expressed in MTC cells, before any drug treatment, at higher pTNM stages (T4), while no expression is observed in lower pTNM stages (T2 and T3) (Ruggeri et al. 2006). This could explain the scarce response rate of MTC patients to chemotherapeutic strategies, based on doxorubicin and cisplatin, in advanced and metastatic tumours. Therefore, the immunohistochemical expression of MDR2 may be useful in clinical practice before applying chemotherapic protocols enclosing doxorubicin and/or cisplatin. Furthermore, we observed a good correlation for the two proteins MDR2 and COX2 with both the pathological and clinical staging criteria. A low MDR2 expression, together with a high COX2 expression were associated with lower pTNM and clinical stages, and may also be considered as possible favourable prognostic markers, specific for early stages of MTC progression. On the contrary, in more advanced stages of MTC, we reported high MDR2 expression and low COX2 expression (Fig. 1D). So, the evaluation of MDR2 and COX2 expression in MTC specimens may improve diagnostic and prognostic evaluation, and contribute to a better characterization and treatment of this type of tumour (Ruggeri et al. 2006).

## Therapy of Thyroid Cancer: How a Biomarker May Become a Therapeutic Target

Because therapeutic options for patients with thyroid cancers that are aggressive and/or do not respond to standard therapies are limited, developing new therapeutic strategies is an important objective of research.

The RET and BRAF kinases and their downstream effectors represent possible targets for novel anticancer therapies.

The RET kinase represents a suitable target for novel drugs helpful in the treatment of both medullary and papillary thyroid cancers, in which activating mutations in the RET proto-oncogene have been identified. Several groups have searched for specific RET kinase inhibitors, able to inhibit autophosphorylation of the receptor, and have published preclinical studies with encouraging results (Carlomagno et al. 2002; Carlomagno et al. 2003; Carlomagno et al. 2006; Petrangolini et al. 2006; Vidal et al. 2005).

Likewise, activating point mutations of B-RAF occur early in the development of PTC, and seem to be related to a more aggressive behaviour in several studies. Clinical evaluation of B-RAF-targeting drugs is undergoing and trials in thyroid cancer are planned. (Fagin, 2004; Ouyang et al. 2006; Salvatore et al. 2006; Mitsiades et al. 2007).

Experimental evidence obtained using inhibitors of uPA and uPAR has validated this system as a therapeutic target for the development of anti-angiogenic and anti-metastatic therapeutic agents. In fact, it has been demonstrated that either inhibition of uPA catalytic activity or prevention of uPA binding to its receptor reduces tumor growth, angiogenesis and metastasis (Mazaar et al. 1999; Duffy, 2004). Thus, uPA and its receptor might represent therapeutic biomarkers also for malignant thyroid neoplasms.

Finally, agents that may induce radioiodine uptake, such as histone deacetylase inhibitors and retinoids, represent another field in new drug development in thyroid cancer (Elisei et al. 2005; Cras et al. 2007).

Potential MTC therapeutic biomarkers are associated with the expression of the MDRs in the tumor cells, as above reported (Ruggeri et al. 2006). Several experimental studies suggest that resistance to chemotherapy of MTC may be circumvented by modulating the expression of MDR1 and/or MDR2, focusing the attention of researchers on MDRs as novel therapeutic targets (Massart et al. 1996; Ratnasinghe et al. 2001; Fagin, 2004; Sorokin, 2004; Zatelli et al. 2005; Pérez-Tomás, 2006; Ruggeri et al. 2006).

## Conclusion

In this review we have evaluated the present knowledge about thyroid tumour markers with a special point of reference to the emergence of new markers arising from the demonstration of several pathways for the development of thyroid malignancy. The analysis of the literature indicates that only few of such thyroid markers may be presently employed in routine clinical practice, while the utility of most of them remain to be ascertained. Certainly, there is a strong need for new cytological and histological markers which could distinguish benign follicular adenomas from FTC. Furthermore, other areas require a careful investigation to look for markers of thyroid cancer recurrence that would serve as early detection systems. However, in the feverish context of the impelling demands to find new decisive markers, it is advisable to be cautious in identifying each new molecule expressed from thyroid cancers as useful in clinical practice.
